# Excess of all-cause mortality is only partially explained by COVID-19 in Veneto (Italy) during spring outbreak

**DOI:** 10.1186/s12889-021-10832-7

**Published:** 2021-04-26

**Authors:** Elisa Gallo, Ilaria Prosepe, Giulia Lorenzoni, Aslihan Şentürk Acar, Corrado Lanera, Paola Berchialla, Danila Azzolina, Dario Gregori

**Affiliations:** 1 Unit of Biostatistics, Epidemiology and Public Health, Department of Cardiac, Thoracic, Vascular Sciences and Public Health, University of Padua, Padua, Italy; 2 Department of Actuarial Sciences, Hacettepe University, Beytepe, Ankara, Turkey; 3 Department of Clinical and Biological Sciences, University of Turin, Turin, Italy; 4 Department of Translational Medicine, University of Oriental Piedmont, Novara, Italy

**Keywords:** COVID-19, Mortality, Italy, Public health burden

## Abstract

**Background:**

Italy has been the first European country to be affected by the COVID-19 epidemic which started out at the end of February. In this report, we focus our attention on the Veneto Region, in the North-East of Italy, which is one of the areas that were first affected by the rapid spread of SARS-CoV-2. We aim to evaluate the trend of all-cause mortality and to give a description of the characteristics of the studied population.

**Methods:**

Data used in the analyses were released by the majority of municipalities and cover the 93% of the total population living in the Veneto Region. We evaluated the trend of overall mortality from Jan.01 to Jun.30. 2020. Moreover we compared the COVID-19-related deaths to the overall deaths.

**Results:**

From March 2020, the overall mortality rate increased exponentially, affecting males and people aged > 76 the most. The confirmed COVID-19-related death rate in the Veneto region between Mar.01 and Apr.302020 is 30 per 100,000 inhabitants. In contrast, the all-cause mortality increase registered in the same months in the municipalities included in the study is 219 per 100,000 inhabitants.

**Conclusions:**

COVID-19 has a primary role in the increase in mortality but does not entirely explain such a high number of deaths. Strategies need to be developed to reduce this gap in case of future waves of the pandemic.

## Background

At the end of February 2020, the first cases of SARS-CoV-2 infection were recorded in Northern Italy. From that moment, the curve of positive cases started growing exponentially [1].

The implemented policies brought to a progressive closure of all the non-essential activities that ultimately resulted in a state of full lockdown on Mar.08 in Northern Italy and on Mar.10 in the entire country.

Although the actions undertaken have shown to be effective in mitigating the progression of the epidemic [2], Italy appears to be the country with the highest proportion of severely ill patients requiring invasive ventilation and with the highest percentage of deaths [3, 4], with the health care system being heavily burdened by the strain of the current epidemic [5]. As observed elsewhere, studying the spread of the outbreak is challenging due to the high number of asymptomatic subjects [6]. In Italy, in addition, different testing policies were implemented across the Italian regions [7], introducing a critical detection bias. Indeed, in some areas only symptomatic/mild symptomatic subjects were tested, whereas in others an extensive testing strategy of both symptomatic and asymptomatic subjects was carried out [7].

Due to such limitations, an evident approach would be to consider overall mortality as a general proxy for the COVID-19 cause-specific deaths. Overall morality trends provide in general useful information [8] and are, more specifically, well-suited to describe epidemics [9–11].

The Veneto region, in Italy, has been one of the first areas to be affected by the SARS-CoV-2 outbreak, and the most aggressive in promoting early detection of cases via mass-screening [2]. In Veneto, COVID-19 cause-specific mortality rates were biased and likely underestimated. Indeed, COVID-19 was both the primary cause of death, but also it might have acted as a mediator, worsening pre-existing conditions, or it simply went undetected under particular circumstances.

In this work, we present estimates of mortality in the first months of the epidemics in a selected number of municipalities in the Veneto region, covering about 93% of the population. In the following section, we evaluate the trend of the overall mortality rate in the Veneto region for both the first semester of 2020 and the last 5 years, to understand the direction and magnitude of change in total mortality in this region and the consequent potential underestimation of the COVID-19 burden.

## Materials and methods

### Data

The Italian National Institute of Statistics (ISTAT) made available on its website the mortality data of 563 Veneto municipalities, with data updated to Jun.30 2020 [12].

The number of municipalities was not planned a priori but represents the number of municipalities that agreed to take part in the project. The municipalities included in the study cover 93% of the Veneto population (4,560,246 out of 4,907,704 total subjects) [12]. The reported characteristics of the subjects are class of age and sex of the deceased. No information is reported about the cause of death.

The number of COVID-19 attributable deaths per region was provided by the Italian Department of Civil Protection and was elaborated by the data warehouse at the University of Padova [13].

### Statistical analysis

We evaluated the trend of the daily all-cause mortality rate in Veneto in the first 6 months of the last 6 years. The regression model for mortality used the observed time series, which was smoothed using local regression, with a smoothing parameter equal to 0.4. Feb.29 was excluded from the analysis.

The all-cause mortality rate for March–April 2019 and March–April 2020 and its change percentage (Mar.01 – Apr.30 2019 vs. Mar.01-Apr.30 2020) was also evaluated overall, by province (as the Veneto region is divided into provinces, in turn divided into municipalities) and by sex. The change percentage in mortality rate was evaluated using the following formula:

$$ \mathrm{Change}\ \mathrm{percentage}=\frac{\ \mathrm{mortality}\ \mathrm{rate}\ \mathrm{in}\ \mathrm{March}-\mathrm{April}\ 2020-\mathrm{mortality}\ \mathrm{rate}\ \mathrm{in}\ \mathrm{March}-\mathrm{April}\ 2019}{\mathrm{mortality}\ \mathrm{rate}\ \mathrm{in}\ \mathrm{March}-\mathrm{April}\ 2019}\times 100 $$

Moreover, all-causes mortality was evaluated in the region by class of age (0–64, 65–75, over 75 years) from Jan.01 to Jun.30 2020.

The COVID-19-related death rate from Mar.01 to Apr.30, when the infections first peaked, was calculated on the entire region per 100,000 inhabitants since COVID-19 data are provided only at the regional level. Moreover, we evaluated the change in mortality rate for March and April 2020 with respect to the average mortality rate for the same months of the last 5 years in the selected municipalities in order to understand if and how the COVID-19 mortality rate can explain the change in all-cause mortality rate.

All the analyses were performed using R 3.6.3 [14].

## Results

**Fig. 1:**
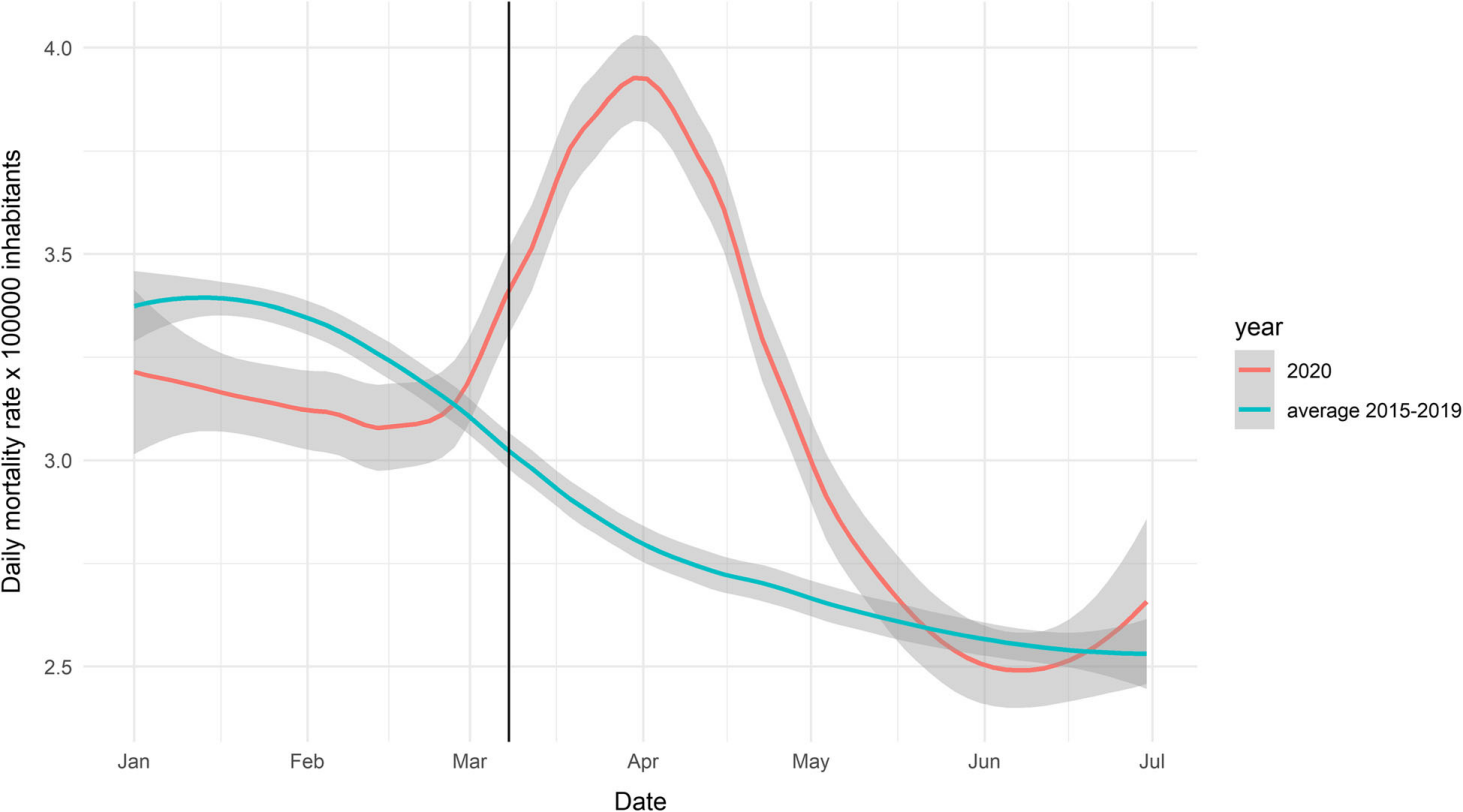
Daily mortality rate per 100,000 inhabitants for the first six months of 2020 and for the same period of the five previous years, obtained through the aggregation of selected municipalities of Veneto. The vertical line corresponds to the beginning of the lockdown

Figure 1 compares the daily mortality rate in 2020 with the average daily mortality rate in the previous 5 years. In January and in the first half of February both values and trends are comparable. Nevertheless, starting from the beginning of March, there is a definite increase in the daily mortality rate in 2020. At the end of March the increase of daily mortality rate starts to slow down and from April the slope starts to decrease. (Fig. 1).

In all provinces, intended as the aggregation by province of the selected municipalities, the overall change from 2019 to 2020 of the mortality rate of March and April is positive, with males showing a higher increase (Table 1). The provinces that were most affected by this early stage of the outbreak are Treviso and Verona. The first case of COVID-19 in Veneto was reported in the province of Padova.

**Table 1:** Mortality change rate from 2019 to 2020 in March and April. Mortality rate in males, females, and overall in 2019 and 2020 stratified by province resulting from the aggregation of selected municipalities

Provinces	Males mortality rate^a^		Females mortality rate^a^		Total mortality rate^a^		Mortality rate variation (%)		
	2019	2020	2019	2020	2019	2020	Males	Females	Total
Verona	1.51	2.30	1.66	2.38	1.59	2.34	51.73	42.91	47.02
Vicenza	1.51	2.01	1.56	2.01	1.53	2.01	33.01	28.92	30.90
Belluno	2.32	2.72	2.43	2.72	2.38	2.72	17.34	11.88	14.47
Treviso	1.46	2.08	1.53	2.07	1.50	2.08	42.56	35.69	38.99
Venezia	1.86	2.21	1.91	2.30	1.89	2.25	18.73	19.95	19.36
Padova	1.61	1.90	1.66	2.16	1.64	2.03	17.75	30.42	24.32
Rovigo	2.03	2.66	2.52	2.80	2.28	2.73	30.76	11.15	19.63

^a^ × 1000 inhabitants

**Fig. 2:**
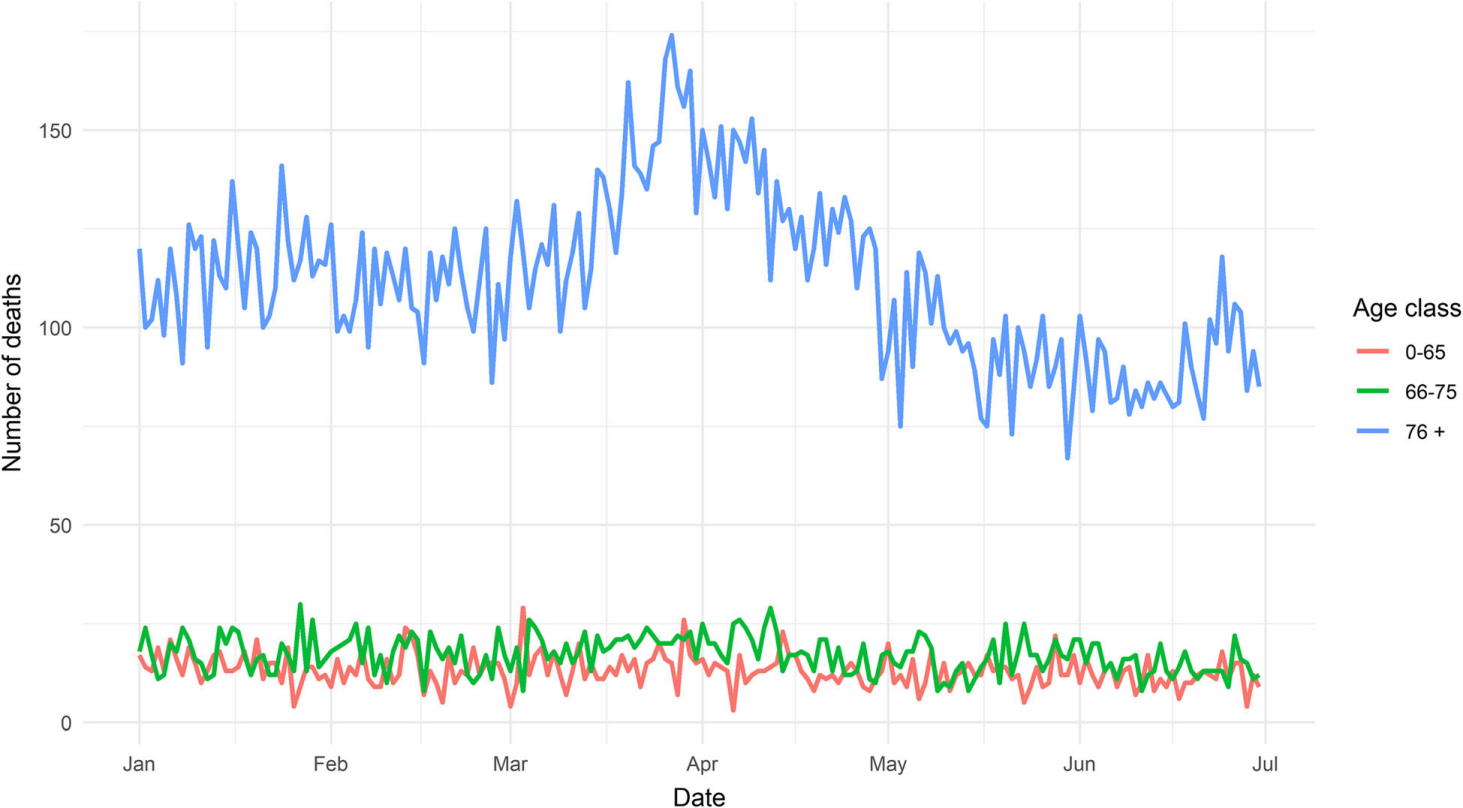
Number of deaths in the Veneto Region resulting from the aggregation of municipalities included in the study from Jan.01 2020 to Jun.30 2020 stratified by classes of age

Figure 2 shows a significant increase in mortality in people over 76 years starting from March, whereas, for people under 65 years old, there is no significant change. The class represented by people from 66 to 75 years old, shows a small increase in the number of deaths, also starting in March.

The confirmed COVID-19-related death rate in the Veneto region between Mar.01 and Apr.30 is 30 per 100,000 inhabitants, whereas, for the municipalities included in the study, the all-cause mortality registered in the same months of 2020 is 219 per 100,000 inhabitants. The average mortality of the five previous years was 173 per 100,000 inhabitants. When adding the COVID-19 mortality to the average mortality of 2015–2019 (203 per 100,000 inhabitants), a 15 per 100,000 inhabitants’ deaths remain unexplained.

## Discussion

The results of this study show an association between COVID-19 and an increase in all-cause mortality in the municipalities of Veneto included in the study.

The Veneto region was the second Italian region to detect COVID-19 positive patients and rapidly responded by introducing restrictive measures to try to reduce the spread of the virus: regional authorities imposed an immediate lockdown to Vo’ [15], the municipality where the first cases were detected; a strong swab campaign, on both symptomatic and asymptomatic individuals, was set up [7]; the use of masks was set as mandatory outdoors as well as indoors. Veneto has been largely studied for the good results obtained in both reducing the spread of the virus and the management of COVID-19 patients [16, 17]. It is indeed interesting to evaluate the trend of mortality (both COVID-19 mortality and all-cause mortality) in a region that was at the forefront of the battle against the virus.

The large increase in the number of positive cases and, consequently, in the number of deaths has been so fast that it can be easily detected as soon as a few days after the identification of the first case [1].

The trend of the daily mortality rate in 2020 seems regular until the end of February and then it suddenly starts to increase rapidly. Comparing this curve with the one given by the average of the 5 previous years, we can notice that the two curves take clearly separate paths starting from March.

In the previous years, the mortality rate starts to slowly decrease from March, whereas in 2020, the trend is the opposite. This change in trend corresponds to the beginning of the COVID-19 outbreak.

Effects of the decree that placed the Veneto Region on lockdown as of Mar 08 start showing after a couple of weeks. Indeed, the daily mortality rate tends to stabilize and then to decrease. The incubation period together with the days from symptoms onset to death could explain this delay in the decrease of the mortality rate [18].

Focusing on 2020, we notice unchanged mortality for people under 65, whereas an increase in the number of deaths in March is evident for 66–75 years older adults and even more for subjects over 65. Usually, the number of deaths in Italy over 65 tends to gradually decrease from the second half of February [19]. However, here the contribution of COVID-19 in increasing the mortality in older people appears evident, and this agrees well with what has already been published [4, 20].

For what concerns gender distribution, males seem to be more prone to death than women. This appears evident for five provinces out of seven and once again agrees with previous studies [4, 21].

The heterogeneity in the mortality rate change observed between the different provinces could be due to many factors, such as: the different healthcare policies adopted; the different geographical accessibility and successibility to the pandemic exposure; the different number of of nursing houses that were highly affected by the pandemic.

The 2020 increase in the death rate in March and April (46 × 100,000) is only partially explained by COVID-19 death rate (30 × 100,000) of the same months, one-third of the deaths remain unexplained.

It is worth pointing out that we analyzed all-cause mortality and not cause-specific mortality. It is likely that the excess of mortality detected is attributable mainly to SARS-CoV-2 infection, including both COVID-19 official deaths and undiagnosed COVID-19 deaths. However, we cannot rule out that such excess of deaths is not directly related to the SARS-CoV-2 infection. As an example, it has been detected a reduction of acute coronary syndrome (ACS) cases referred to the emergency departments after the beginning of the outbreak [22]. Such reduction would be attributable to not only an actual reduction of ACSs, but also to the fear of contamination, which makes people in need of health care more reluctant to visit the hospital, and to the difficulties in accessing healthcare. A survey conducted across the six continents (with America divided into Northern and Southern) just highlighted that issue by showing a delay in time to admission of STEMI patients (ST-elevation myocardial infarction) in countries with full lockdown, the same that was imposed in the Veneto region [23]. Together with these difficulties in accessing the healthcare system, increased systemic inflammation has been observed in subjects undergoing routine hematology tests during the lockdown [24]. The combination of all these psychological and pathological aspects could play a role in the increase in mortality observed. The experience gained during the first wave of COVID-19 will be useful to be ready for future waves of the pandemic.

### Study limitations

ISTAT only provides the number of all-cause deaths and the number of residents. This reflects in the modeling approach, which considers only gross mortality rates since no information is available about the primary cause of death or about the possible drug therapy subjects are undergoing or other personal characteristics. Moreover, data on COVID-19 related deaths did not include subjects’ characteristics, thus not allowing the comparison with overall mortality by age group or sex.

## Conclusions

The current data show increased mortality in older people and a greater change in mortality in male subjects. The curve of observed mortality slowed down starting from April, proving that the lockdown measures worked, but the number of COVID-19 deaths in Veneto does not seem to match the real amount of subjects that have died in the municipalities included in the study. If such data is confirmed, it will become clear that the COVID-19 outbreak public health burden is even worse than expected because it would be associated with an excess of mortality not directly related to the infection.

Continuous surveillance, enforced in every municipality, must be conducted in the coming weeks to evaluate the trend of COVID-19, improve knowledge on this matter, and act conscientiously.

## Data Availability

The public access to the databases is open. The datasets generated and analysed during the current study are available in the mortality_veneto repository, https://github.com/UBESP-DCTV/mortality_veneto. The datasets can be found as .rds files in the raw-data folder of the repository, https://github.com/UBESP-DCTV/mortality_veneto/tree/master/raw-data

## Abbreviations

SARS-CoV-2: Severe acute respiratory syndrome - coronavirus - 2
COVID-19: Coronavirus disease 2019
ISTAT: Italian national institute of statistics
ACS: Acute coronary syndrome
STEMI: ST-elevation myocardial infraction
